# Genome-Wide Identification of m^6^A Writers, Erasers and Readers in Poplar 84K

**DOI:** 10.3390/genes13061018

**Published:** 2022-06-05

**Authors:** Xiaochen Sun, Wenli Wu, Yanfang Yang, Iain Wilson, Fenjuan Shao, Deyou Qiu

**Affiliations:** 1 State Key Laboratory of Tree Genetics and Breeding, Key Laboratory of Tree Breeding and Cultivation of National Forestry and Grassland Administration, Research Institute of Forestry, Chinese Academy of Forestry, Beijing 100091, China; sxchen@caf.ac.cn (X.S.); wwlarroyo@163.com (W.W.); echojjf@163.com (Y.Y.); qiudy@caf.ac.cn (D.Q.); 2 CSIRO Agriculture and Food, Canberra, ACT 2601, Australia; iain.wilson@csiro.au

**Keywords:** m^6^A modification, writers, erasers, readers, *LBD 15*, poplar 84K

## Abstract

N^6^-methyladenosine (m^6^A) RNA modification is a conserved mechanism to regulate gene expression that plays vital roles in the development of plants. However, the m^6^A RNA modification in forest trees remains limited. Here, we performed a complete analysis of m^6^A writers, erasers and readers in Poplar 84K, including gene location, gene structures, conserved motifs, phylogenetic relationships, promoter analysis, expression profiles and the homology modeling. We have identified 61 m^6^A pathway genes in Poplar 84K (*Populus alba × Populus glandulosa*), including 14 m^6^A writers, 14 m^6^A erasers and 33 m^6^A readers. Phylogenetic analysis indicated that the m^6^A writers and erasers were clustered into four groups and m^6^A readers were clustered into two groups. Promoter analysis showed that m^6^A pathway genes were mainly responsive to low oxygen followed by ABA and ethylene. The expression of the identified m^6^A pathway genes showed tissue-specific expression patterns in leaves, xylem, phloem and roots. Moreover, 17 genes were significantly up-regulated and 13 genes were significantly down-regulated in poplar overexpressing the transcription factor *LBD15*. Homology modeling and molecular docking results suggested that *PagFIP37b* was most likely to be regulated by *LBD15*, and the qPCRshowed that *PagFIP37b* were up-regulated in the *LBD15-oe* plants. The results provide insights that aid in the future elucidation of the functions of these m^6^A pathway genes and the epigenetic regulation mechanism of these genes in Poplar 84K.

## 1. Introduction

The RNA epigenetic modification plays a significant role in the regulation of gene expression [1,2]. Up to now, more than 100 modifications have been reported in RNA [3], and N^6^-methyladenosine (m^6^A) RNA methylation is the most abundant intermediate chemical modification of post-transcriptional gene regulation in eukaryotes. The m^6^A modification occurs at the sixth N atom of adenine, and the m^6^A accounts for up to 80% of RNA methylation modifications in eukaryotic cells and 50% of methylated modifications in mRNA [4]. m^6^A is widely studied in eukaryotes species, such as yeast, plants, flies, mammals and viral RNAs with a nuclear phase [5].

m^6^A modification affects almost every stage of mRNA metabolism, as it provides a binding site for effector proteins that regulate the stability, splicing and translation of mRNA [6,7]. Liu and Pan confirmed that the m^6^A could recognize RNAs all the time through identifying the m^6^A responsive RNA-binding protein [8]. The core proteins that participate in the m^6^A pathway are divided into three groups named writers (methyltransferases), erasers (demethylases) and readers [6,9]. In mammals, METTL3 (methyltransferase-like 3), METTL14 (methyltransferase-like 14) and WTAP serve as writers, FTO belongs to erasers, the readers included ALKBH5 and YTH (YTH domain family 2) [6,10,11]. In plants, the MTA (homologue of human METTL3), MTB (homologue of human METTL14), VIR, HAKA, and FIP (ortholog of human WTAP) were identified as plant m^6^A writers [5,7,12,13,14]. ALKBH9B and ALKBH10B proteins were considered as erasers to remove the methylation modification in the nucleus [5,9,10]. The reader proteins mainly included ECT2/3/4 and CPSF30 [6,9]. m^6^A has been examined in many species, including *Arabidopsis* [11], maize [12], wheat [13], oat [14], rice [15], sea buckthorn and apple [16,17]. Recently, the function of some m^6^A pathway genes in plants has been studied. The inactivation of MTA could reduce m^6^A modification and lead to a failure of embryonic development and reduced apical dominance [11,18]. Shoot meristems require FIP37 to be maintained and are continuously produced in *Arabidopsis* [19]. ECT2/3 are necessary to regulate the formation and timing of leaf morphogenesis [6]. These studies indicated that the m^6^A modifications in mRNA play a crucial role in plant development.

m^6^A RNA methylation is a conserved mechanism to enrich and control gene expression and plays an important role in organisms. The distribution pattern of methylation sites and the consensus sequence of m^6^A seem to be conserved in human and yeast; both the modification sites are enriched at the 3′untranslated regions (3′UTRs). In addition, m^6^A in human is also enriched around stop codons and within internal long exons [5]. However, plants may have evolved with unique mechanisms in m^6^A methylation machinery. In plants, it has been shown that m^6^A is usually enriched around the stop codon, 3′UTRs, long exons, TSS (the transcription start site) and TES (transcription end site) [16,20,21,22]. The writers in *Arabidopsis* could recognize the consensus motif RRACH [23]. However, not all RRACH motifs in plants are associated with m^6^A modification, and the molecular mechanism of recognition is also undefined [8].

The m^6^A level was affected by the expression of m^6^A pathway genes. It has been shown that the m^6^A levels were reduced through down-regulation of the m^6^A pathway genes [23]. However, knowledge of RNA modification in forest trees remains limited. Poplar 84K has become a model plant for forest tree studies, since it is a fast-growing poplar with an available whole genome sequence, high transformability and economic and ecological value. In our previous studies, we have reported that an ortholog gene of *AtLBD15* from Eucalyptus grandis takes part in the leaf development in Poplar 84K [24] and analyzed the differential m^6^A modification sites between Poplar 84K and the *LBD15* overexpression plants [22]. In order to further elucidation of the molecular mechanism of m^6^A modification associated with *LBD15*, here, we systematically identified m^6^A writers, erasers and readers genome-wide in Poplar 84K. The gene structures, gene location, conserved motifs and phylogenetic relationships were identified, and the homology modeling of these proteins and LBD15 were analyzed. In addition, the tissue-specific expression profiles and the expression of these genes in Poplar 84K and the *LBD15* overexpression plants were also investigated. The results provide insight to further elucidate the functions of the genes of m^6^A pathway and the epigenetic regulation mechanism of m^6^A in Poplar 84K.

## 2. Materials and Methods

### 2.1. Plant Materials

Poplar 84K was planted in the greenhouse located at the Chinese Academy Forestry, Beijing, China. Leaves, xylem, phloem and roots were sampled from 6-month-old plants and stored in liquid nitrogen for RNA isolation. The shoots cultivated for 6 weeks were collected from Poplar 84K and *LBD15* overexpression plants and stored in liquid nitrogen until use. Three independent biological replicates were performed from three whole plants for each sample.

### 2.2. Identification of m^6^A Pathway Genes in Poplar 84K

All the m^6^A pathway genes from *Arabidopsis* and *Oryza sativa* protein sequences were downloaded from GenBank (Table S3) and used as a query to search for homologous genes in Poplar 84K genome through tBLASTn with an e-value of 1 × 10^−5^ [25]. The conserved domains of the candidate genes were analyzed in NCBI-CDD [26]. The molecular weight (Mw) and isoelectric point (pI) of these proteins were investigated using the ExPASy online software [27]. Their subcellular localization was predicted based on the Busca online software [28].

### 2.3. Phylogenetic Tree, Gene Structure, Conserved Motifs, Promoter Prediction and Protein Interaction Analysis

The chromosomal location and gene structure of these m^6^A pathway genes were obtained from the genome annotation files, which were downloaded from (https://www.ncbi.nlm.nih.gov/genome/87686, accessed on 1 June 2022). The chromosome physical location of those genes was displayed by MapGene2Chromosome v2.0 [29], and the gene structure was shown using a Gene Structure Display Server [30]. The MEME tool was used to predict the conserved motif of the candidate protein sequences [31]. The maximum motif number was set to 5, and other parameters were left on the default settings. The phylogenetic trees and dN/dS analyzed were constructed by MEGA7.0 software [32] using the muscle and neighbor-joining (NJ) method with the bootstrap value of 1000 [33]. The upstream 2000 base pair (bp) genomic DNA sequences from the transcription start sites of m^6^A pathway genes were predicted in PlantCare database [34] to identify the putative cis-regulatory elements.

### 2.4. Quantitative Real-Time Reverse Transcription-PCR (qRT-PCR)

Total RNA of leaves, roots, xylem and phloem from Poplar 84K and *LBD15-oe* plants were isolated by RNA Easy Fast Plant Tissue Kit (TIANGEN, Beijing, China). The cDNA was generated by reverse transcription using FastKing RT Kit (TIANGEN, Beijing, China). The qRT-PCR analysis of m^6^A pathway genes were performed with SYBR^®^ rapid quantitative PCR Kit (KAPA KK4601, Pleasanton, CA, USA) using the methods described previously [35]. Pagactin was used as a reference [24]. The primers of all the genes were listed in Table S5, and the results were analyzed using the 2^−ΔΔCt^ method [24].

### 2.5. Homology Modeling of 3D Structures, Molecular Docking and Protein Docking

The 3D structures of m^6^A pathway proteins are important for investigating their gene function. The 3D structure of m^6^A pathway proteins and LBD15 were predicted using the homology modeling method. The PDB database [36] was used to find the best template, and the model was built by Swiss-Model [37]. The predicted cis-elements of m^6^A pathway proteins that can interact with LBD15 were performed by JASPAR [38]. The highest scoring cis-element was used to dock with LBD15 through Autodock vina [39]. The protein docking of PagMT families was performed using ZDOCK [40], and PDBePISA [41] was used to analyze the docking results. Pymol software was used for evaluation of quality, and the equation linking affinity (Ka) and ligand free energy of binding ΔGwere calculated by formula:ΔG = −RTln (K_A_/C) (T is temperature in kelvin, C is 1 M concentration and R = 8.314 J/mol/K) [42].

### 2.6. Statistical Methods

In this study, one-way ANOVA was used to perform the statistical analysis for the differential tissues expression of m^6^A pathway genes through IBM SPSS 19 software. We ranked all averages from highest to lowest and marked the letter a after the highest average; then, the mean was compared with the following means, and any mean that was not significantly different was marked with the letter a, while any mean that was significantly different was marked with the letter b, and so on until the smallest average has a marked letter and stops. Moreover, the statistical analysis of the expression between Poplar 84K and *LBD15*-oe plants were calculated by T-test was using IBM SPSS 19 software. *p* < 0.05 (*) was considered statistically significant, while *p* < 0.01 (**) was considered extremely significant.

## 3. Results

### 3.1. Genome-Wide Identification of m^6^A Pathway Genes in Poplar 84K

The protein sequences involved in the m^6^A pathway in plants including *Arabidopsis* and *O.sativa* were downloaded from GenBank. tBLASTn analysis of these protein sequences against the genome of Poplar 84K were performed to identify putative m^6^A pathway genes. As a result, a total of 61 m^6^A pathway-like genes were identified in Poplar 84K, including 14 m^6^A erasers (14 PagALKBHs), 14 m^6^A writers (8 PagMTs, 2 PagFIP37s, 2 PagVIRILIZERs and 2 PagHAKAIs), 33 m^6^A readers (28 PagECTs and 5 PagCPSF30s). Analysis of gene feature showed that the length of the ORF (open-reading frame) varied from 1783 to 7182 bp of m^6^A erasers, 1285 to 17,347 bp of m^6^A writers, and 3379 to 16,254 bp of m^6^A readers, respectively. The amino acid length varied from 240 to 770 aa for m^6^A erasers, 288 to 2179 aa for m^6^A writers, and 398 to 975 aa for m^6^A readers, respectively. The average molecular weight (Mw) is 44.41, 82.22 and 69.89 kDa, respectively, and the theoretical pI of m^6^A pathway genes ranged from 4.62 to 9.22. The prediction of subcellular localization displayed that 59 genes were located in the nucleus, and another 2 genes were located in cytoplasm (Table 1).

### 3.2. Phylogenetic Analysis of m^6^A Pathway Genes in Poplar 84K

**Figure 1:**
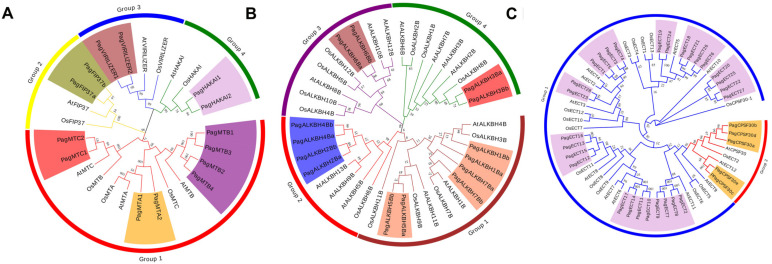
Phylogenetic trees of m^6^A pathway genes from Poplar 84K, *A.* *thaliana* and *O. sativa* (**A**) Writers; (**B**) Erasers; (**C**) Readers. The phylogenetic trees were constructed using MEGA 7.0 by the neighbor-joining (NJ) method with 1000 bootstrap replicates. The groups of m^6^A pathway genes from Poplar 84K are shown in different colors.

To investigate the phylogenetic relationship of m^6^A pathway genes in Poplar 84K, the phylogenetic tree was constructed using the protein sequence of the N^6^-methyladenosine writers, erasers and readers in Poplar 84K with the corresponding genes in *Arabidopsis* and *O. sativa*, respectively. The results showed that the writers in Poplar 84K were clustered into four groups, including PagMT, PagFIP37, PagVIRILIZER and PagHAKAI families (Figure 1A). The PagMT group was divided into three subgroups: PagMTA, PagMTB and PagMTC. The erasers in Poplar 84K only contained the PagALKBH family, which was clustered into four groups (Figure 1B). The erasers in Poplar 84K had a distribution in each group. The phylogenetic analysis showed that the readers in Poplar 84K were classified into two groups, PagECT and PagCPSF30 (Figure 1C). PagECT family contained a conserved YTHDF domain, whereas the PagCPSF30 family contained a conserved YTHDC domain. This is consistent with previous studies from other plants, suggesting that the readers have a conserved role in the plants.

The dN/dS value of the m^6^A pathway genes were calculated by the MEGA7.0 software to detect molecular selection effects (Table S1). As a result, the m^6^A pathway genes except for PagCPSF30s had a dN/dS < 1, indicating that they had undergone strong purifying selection. There were both dN/dS > 1 and dN/dS < 1 in the PagCPSF30 family, suggesting it had undergone purifying selection and position section. The results suggested that the m^6^A pathway genes were highly conserved during the evolutionary process with the exception of PagCPSF30s.

### 3.3. Analysis of Chromosomal Location

**Figure 2:**
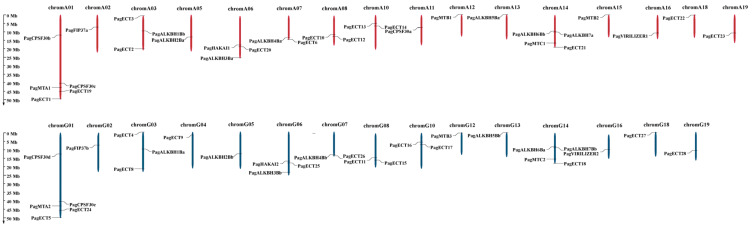
Genes location on chromosomes. The chromosomes in genome A are shown in red, and the chromosomes in genome G are shown in blue.

To further understand the evolutionary relationship of the 61 m^6^A pathway genes, the chromosomal location of these genes was determined. The result showed that these genes were distributed on 31 chromosomes (Figure 2). Among them, chromA01 and chromG01 contain the largest number of m^6^A pathway genes; *PagCPSF30b*, *PagCPSF30c*, *PagMTA1*, *PagECT1* and *PagECT19* were located on chromA01. Additionally, *PagCPSF30d*, *PagCPSF30e*, *PagMTA2*, *PagECT5* and *PagECT24* were also located on chromG01. ChromA14 and chromG14 contained four m^6^A pathway genes, respectively. *PagALKBH6Bb*, *PagALKBH7Ba*, *PagMTA3* and *PagECT21* were located on chromA14, and *PagALKBH7Bb*, *PagALKBH6Ba*, *PagMTA4* and *PagECT18* were located on chromG14. The result suggested that these genes were generated by segmental duplication, which was the single driving force in the evolutionary process of m^6^A pathway genes in Poplar 84K.

### 3.4. Gene Structure and Conserved Motif Analysis

**Figure 3:**
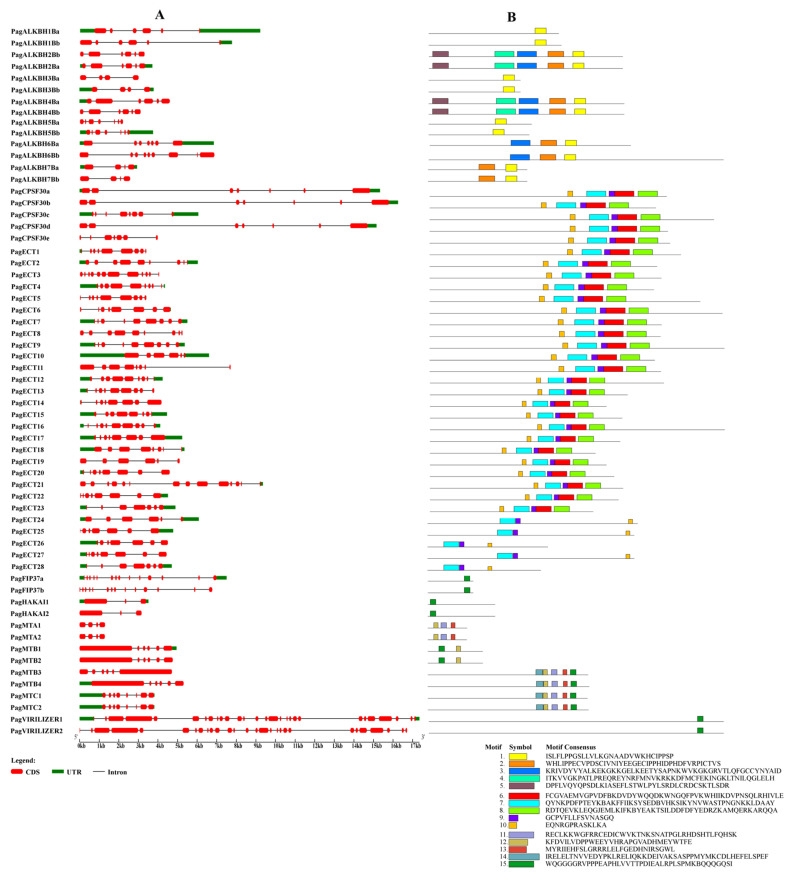
The gene structure and conserved motifs of m^6^A pathway genes. (**A**) Exons, UTRs, introns and intron phases are shown. (**B**) Motifs are represented by boxes.

The exon–intron pattern is an important feature for a gene and can provide important evidence for gene functional diversification, so the exon–intron patterns of m^6^A pathway genes were determined (Figure 3). The intron numbers in writers varied from 2 to 27. For erasers, the intron number varied from 3 to 6. The intron number of the readers clustered in the PagECT group varied from 5 to 11, and those clustered in PagCPSF30 group varied from 5 to 7. The PagCPSF30 gene family has the largest intron size. The results showed that the genes clustered into the same clade in the phylogenetic tree had similar exon–intron patterns, indicating the conservation of these genes during evolution.

The conserved motif analysis results showed that a total of 15 motifs were identified in the m^6^A pathway genes in Poplar 84K and the genes clustered with the same group had similar motifs, which is consistent with the results of the gene structure and the phylogenetic tree. We found that some motifs were highly conserved in the m^6^A pathway genes in Poplar 84K; for example, the motif 1 was found in all the erasers, motif 7, motif 9 and motif 10 were present in all the readers and motif 15 was present in all writers.

### 3.5. Promoter Analysis of m^6^A Pathway Genes

**Figure 4:**
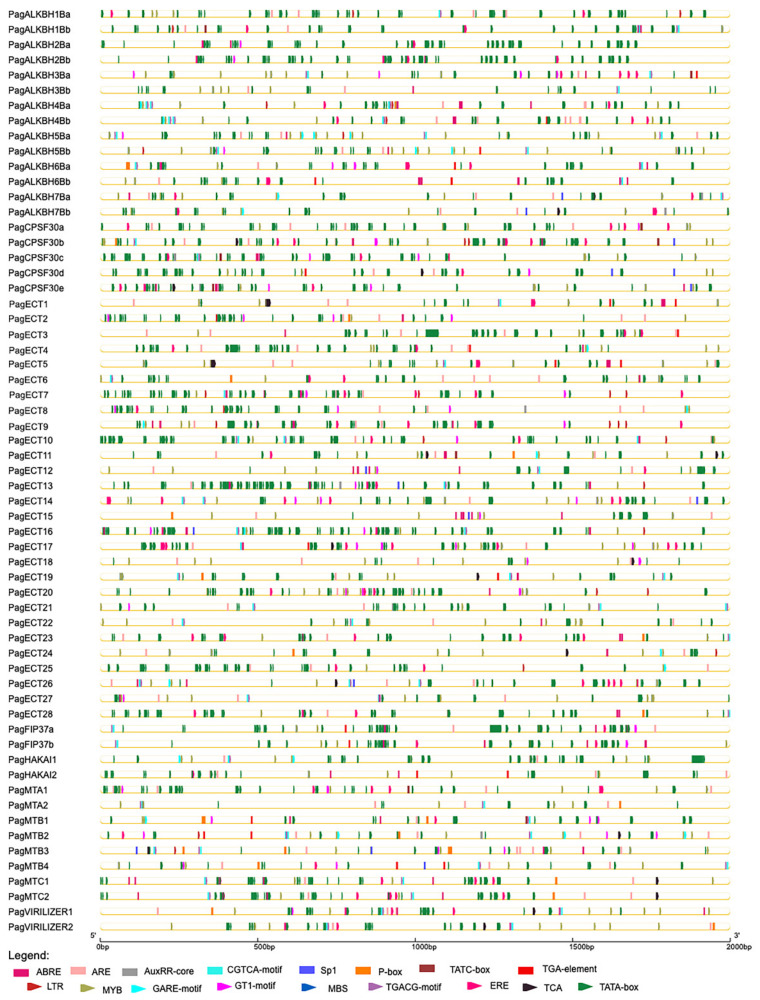
The *cis*-regulatory elements in the promoter of 61 m^6^A pathway genes. The *cis*-regulatory elements were represented bytriangle and rectangle in different colors.

The *cis*-elements of these genes are shown in Figure 4 and Table S2. CAAT-box and TATA-box were found in all the m^6^A pathway genes. The main *cis*-acting elements were predicted to respond to abiotic stress, hormones and inducers such as methyl jasmonate (MeJA), light, anaerobic, ethylene, salicylic acid (SA), drought, low temperature, abscisic acid (ABA), gibberellin and auxin. The largest number of *cis*-elements were light-response elements, and they were found in all the m^6^A pathway genes. For erasers, 13 genes had ABA response elements, while 12 genes had anaerobic response and ethylene response elements. In readers, 33 genes have anaerobic response elements. In writers, anaerobic response elements were found in all the members. The results showed that m^6^A pathway genes were mainly responsive to low oxygen followed by ABA and ethylene.

### 3.6. Expression Profiles of m^6^A Pathway Genes

**Figure 5:**
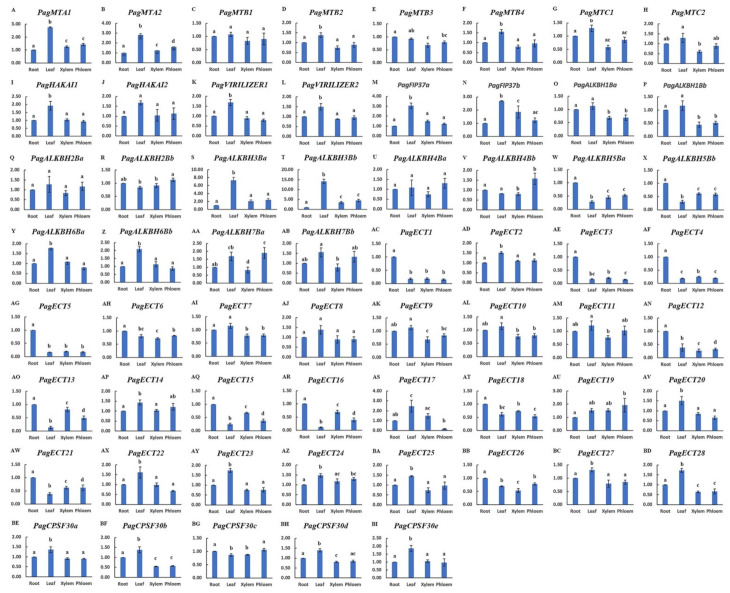
qPCR validation of 61 m^6^A pathway genes in tissues. Figure (**A**)–(**BI**) showed the expression level of 61 genes in differential tissues. One-way ANOVA was calculated using IBM SPSS 19 software. The a, b, c and d indicated whether the difference was significant. The same letter marked in the same gene among different tissues indicated no significant difference, and different letters indicated significant difference.

The expression of m^6^A pathway genes in differential tissues was investigated using qPCR (Figure 5). The results showed that the expression of these genes displayed differential expression patterns. All the writer genes were highly expressed in leaves, especially, the PagFIP37 genes which had the highest expression in leaves. The readers that clustered into the same subgroup displayed similar expression patterns; for instance, *PagECT1*, *PagECT3*, *PagECT4* and *PagECT5* were mainly expressed in roots, while *PagECT20*, *PagECT22*, *PagECT25* and *PagECT27* showed the highest expression levels in leaves. *PagECT19* had the highest expression level in the phloem. As for erasers, the different erasers showed divergent expression patterns, such as *PagALKBH4s* and *PagALKBH5s*, which were mainly expressed in phloem, and *PagALKBH3s* showed significantly high expression in leaves.

**Figure 6:**
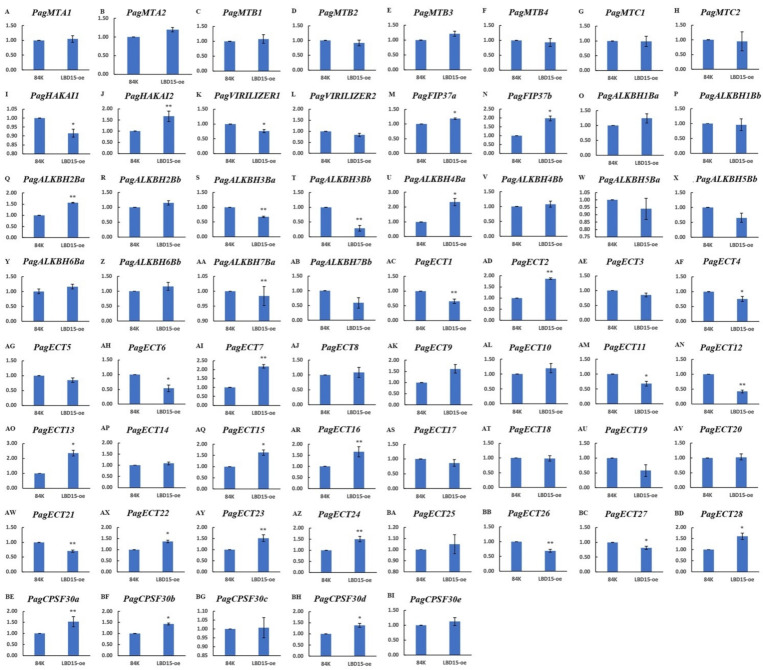
qPCR validation of 61 m^6^A pathway genes in Poplar 84K and LBD15-oe plants. Figure (**A**)–(**BI**) showed the expression level of 61 genes in Poplar 84K and LBD15-oe plants. A *t*-test was calculated using IBM SPSS 19 software. * represent *p* < 0.05 and ** represent *p* < 0.01.

The expression of the m^6^A pathway genes in Poplar 84K and *LBD15-oe* plants was also investigated by qPCR (Figure 6). The results showed that the expression of the m^6^A pathway genes was regulated in the *LBD15-oe* plants. There were 17 genes that were significantly up-regulated; among them, the expressions of *PagALKBH4Ba, PagECT7* and *PagECT13* were up-regulated more than 2-fold, while *PagHAKAI2*, *PagFIP37b*, *PagALKBH2Ba*, *PagECT2*, *PagECT9*, *PagECT15*, *PagECT16*, *PagECT23*, *PagECT28* and *PagCPSF30a* were up-regulated more than 1.5-fold in the *LBD15-oe* plants. Conversely, 13 genes were significantly down-regulated; for example, *PagECT12* and *PagALKBH3Bb* were down-regulated more than 2-fold in the *LBD15-oe* plants. The results implied that the regulatory role of m^6^A modification may be associated with the expression of *LBD15*.

### 3.7. Homology Modeling, Molecular Docking and Protein Docking

**Figure 7:**
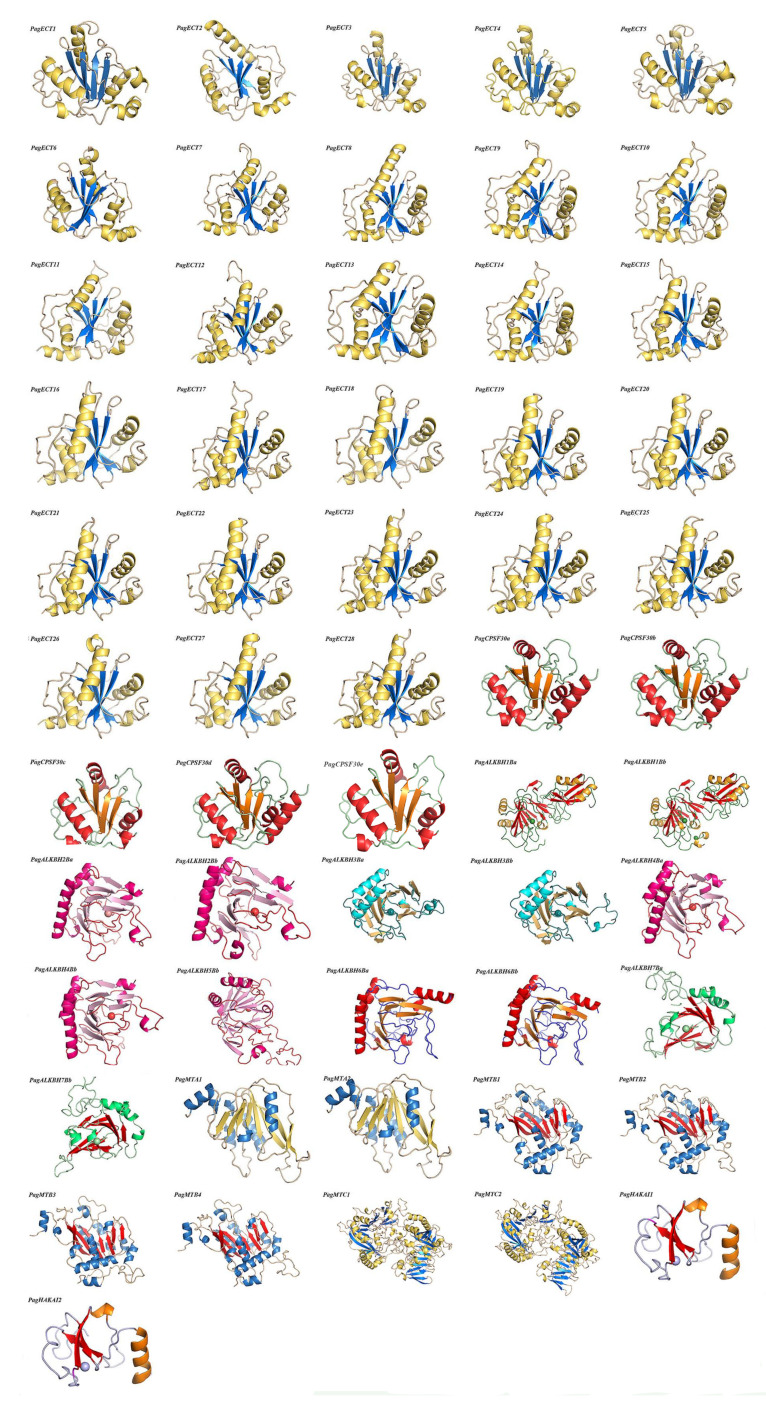
The homology modeling of m^6^A pathway genes in Poplar 84K. The α-helix, β-fold and random coil are shown, and the ball representsmetal ions.

**Figure 8:**
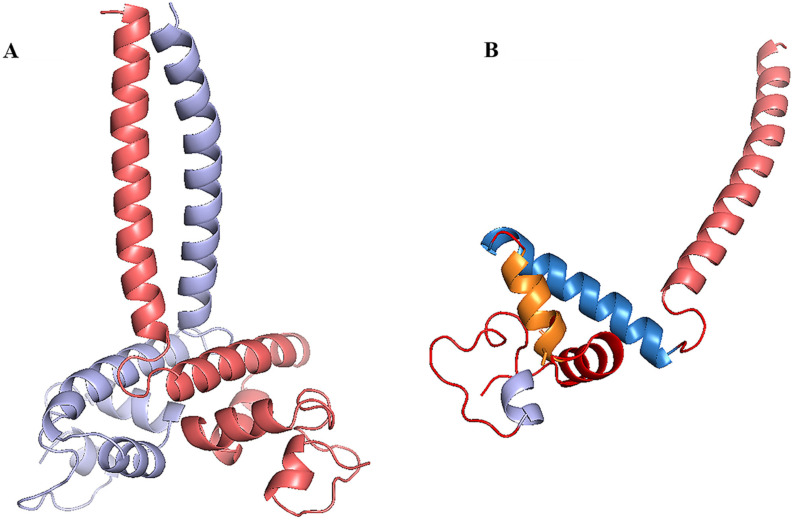
(**A**) The homology modeling of LBD15. (**B**) The structure of chain A in LBD15.

The protein model of the m^6^A pathway genes and LBD15 were built by homology modeling, as shown in Figure 7 and Figure 8A. The template and RMSD are shown in Table S3. The RMSD (root-mean-square deviation) is less than 1Å with respect to the templates, suggesting that the homology modeling was reliable. As a result, we obtained the 3D structure of a total of 56 proteins of the m^6^A pathway genes; five other proteins were not modeled, as there was no homologous template to build in the database. In the 3D structures, the α-helix, β-fold and random coil were signed in different colors. All the structures of readers contained five α-helixes and four adjacent β-folds and formed highly similar spatial structures. The 3D structures of erasers varied among groups based on the metal ions; for example, PagALKBH1Ba contained one Mn ion, while PagALKBH1Bb contained one Mn ion and one Zn ion in the structure, and PagALKBH7Bs and PagALKBH3Bs contained one Fe ion. For the writers, PagMTCs displayed as dimers and contained two centrosymmetric monomeric proteins. The proteins that clustered into the same group had highly similar 3D structures, indicating that these genes were highly conserved in the plants. 

The LBD15 protein contains a typical LOB domain displayed as dimers, which contained two centrosymmetric monomeric chains (chain A and chain B) (Figure 8A), and the monomeric chain consisted of five α-helixes (Figure 8B). The most reliable *cis*-elementin promoter of the m^6^A pathway genes that up-regulated more than 1.5-fold in the LBD15-oe plants were predicted, and the molecular docking of these *cis*-elements with LBD15 was performed. The results of *cis*-elements docking with LBD15 are shown in Table 2 and Figure S1. Among those 13 genes, PagFIP37b could be the most reliable to interact with LBD15 according to the Affinity.

The protein docking of PagMTs was performed. The results of protein docking are shown in Table 3; the docking of PagMTA1-PagMTC1 and PagMTA2-PagMTB1 are the most credible based on the score of ΔiG (kcal/M) and *p*-value. The results revealed that PagMTA-PagMTB, PagMTA-PagMTC, and PagMTC-PagMTB could bind and formed a dimer, which could help us understand how these writers work.

## 4. Discussion

m^6^A RNA methylation is the most abundant intermediate chemical modification of post-transcriptional gene regulation in eukaryotes. It is indispensable for plant growth and development through participating in the mRNA splicing, stability and translation [6,7]. In our previous study, the regulation of the m^6^A modification between Poplar 84K and the *LBD15* overexpression plants was analyzed [22]. In order to increase our understanding of the mechanism of m^6^A modification and the epigenetic regulation of *LBD15*, here, we systematically at a genome level identified 61 m^6^A pathway genes in poplar 84K, including 14 m^6^A erasers, 14 m^6^A writers and 33 m^6^A readers. The gene structures, gene location, conserved motifs and phylogenetic tree were performed; the tissue-specific expression profiles and the expression of these genes in Poplar 84K and the *LBD15* overexpression plants were investigated. In addition, the 3D structures and protein docking of the identified proteins were also analyzed. Our results provide insight into understanding the roles of these m^6^A pathway genes and the epigenetic regulation mechanism of these genes in Poplar 84K.

In this study, in total, we identified 61 m^6^A writers, erasers and readers in Poplar 84K; genome-wide, the number of the m^6^A pathway genes was significantly greater than the number found in *O.sativa* and *Arabidopsis*, which both contain 28 genes. This may be partially explained by the fact that Poplar 84K is a hybrid of *P.alba* and *P.glandulosa*. It has two subgenomes, which have different gene numbers based on the chromosomal localization analysis. Based on the phylogenetic tree, the 14 m^6^A writers in Poplar 84K were clustered into four groups, implying that the four types of writers have different functions. Among them, the PagMT group was the largest one, which contained eight members. This is distinct from its counterparts in *Arabidopsis*, suggesting that the PagMT group proteins potentially have more functions in Poplar 84K. The eraser is a demethylase which could remove the m^6^A modification in the nucleus. In Arabidopsis, the functions of *ALKBH9B* and *ALKBH10B* are well studied [5,9,10]. Six ALKBH proteins in Poplar 84K were clustered into the same clades with AtALKBH9B and AtALKBH10B, including PagALKBH2Ba, PagALKBH2Bb, PagALKBH4Ba, PagALKBH4Bb, PagALKBH6Ba and PagALKBH2Bb, indicating they may have a role similar to m^6^A demethylation. The 33 readers were classified into two groups according to the YTHDC domain and YTHDF domain; the result is consistent with other plants [25], suggesting that the readers are highly conserved in plants.

The m^6^A modification controls gene expression through its writers, readers and erasers, so the expression of the writers, readers and erasers is very important for investigating their potential functions. In this work, the expression of m^6^A pathway genes in four tissues including leaves, roots, xylem, and phloem was analyzed. The results revealed that all the identified genes were detected in the leaves, roots, xylem, and phloem of Poplar 84K, but they displayed differential expression profiles. The genes clustered into the same clade showed a similar expression pattern, suggesting they may play similar roles in the plant growth and development. Our previous study has reported that an ortholog of *AtLBD15* is involved in the development of leaves [24]. We found that the level of m^6^A modification in *LBD15* overexpression plants was altered. In order to further understand the mechanism of m^6^A modification in LBD15 overexpression plants, we detected the specific expression of m^6^A pathway genes in CK and *LBD15-oe* plants. As a result, we found that the expressions of the m^6^A pathway genes were regulated in the LBD15-oe plants. For example, some readers including *PagECT7*, *PagECT13, PagECT15*, *PagECT16*, *PagECT2*, *PagECT9*, *PagECT23*, *PagECT28* and *PagCPSF30a* were up-regulated more than 1.5-fold in *LBD15-oe* plants. Moreover, these genes were mainly expressed in leaves, suggesting these genes are probably involved in the leaf development. It has been shown that proteins ECT2/3/4 are essential for leaf formation in *Arabidopsis* [8], which is consistent with our results. On the contrary, *PagHAKAI2* was significantly down-regulated in the *LBD15-oe* plants; this is consistent with our previous work, which showed that 4260 down-regulated m^6^A peaks were detected in *LBD15-oe* plants [22]. Taken together, the level of m^6^A in the *LBD15-oe* plants was affected by *LBD15* through regulating the expression of m^6^A pathway genes.

The m^6^A in the plants is a complex process; the functions of the m^6^A writers, erasers and readers in plants are still unclear [8]. The homology model and molecular docking could help us better understand the 3D structure of proteins and drive our future research. The results of molecular docking between the cis-element of m^6^A pathway genes and *LBD15* showed that *PagFIP37b* could be regulated by LBD15, which will provide a direction for studying the epigenetic regulation mechanism associated with LBD15 in Poplar 84K. In *Arabidopsis*, it has been shown that MTA and MTB could interact with each other and form homodimers [43]. In this study, the protein–protein docking of PagMTs revealed that PagMTA-PagMTB, PagMTA-PagMTC, and PagMTC-PagMTB could bind and form a dimer. The ΔiG < 0 indicated that the docking was reliable [41]. The *p*-value < 0.5, implying the interface surface can be interaction-specific. The results showed that two PagMTs could form a dimer, which is consistent with *Arabidopsis*, suggesting this prediction was reliable. These results will provide valuable information for further study of the functions of the m^6^A pathway genes in the Poplar 84K.

## 5. Conclusions

In this study, a thorough and systematic approach, combining phylogenetic analysis, chromosomal localization, gene structure, conserved motif and promoter analysis as well as expression and 3D structures, was performed to help characterize the 61 m^6^A pathway genes identified in the genome of Poplar 84K. The results revealed that the m^6^A pathway genes in Poplar 84K were highly evolutionary conserved. The expression of the identified m^6^A pathway genes showed tissue-specific expression patterns in leaves, xylem, phloem and roots. These genes were regulated in the *LBD15-oe* plants. The results of 3D structures docking showed that PagFIP37b could be the most reliable to regulate by LBD15 and two PagMTs could form a dimer, which could help us understand how writers work. Our results provide some insight into the functions of these m^6^A pathway genes and the epigenetic regulation mechanism of these genes in Poplar 84K.

## Figures and Tables

**Table 1:** Sequences feature of m^6^A pathway genes in Poplar 84K.

Genes	Length (bp)	Length (aa)	PI	MW (kDa)	Subcellular Location
PagALKBH1Ba	6018	340	6.41	37.91	nucleus
PagALKBH1Bb	7182	346	6.51	38.59	nucleus
PagALKBH2Ba	3684	506	5.62	56.68	nucleus
PagALKBH2Bb	3280	506	5.72	56.74	nucleus
PagALKBH3Ba	2086	240	9.17	27.97	nucleus
PagALKBH3Bb	2646	240	9.22	28.03	nucleus
PagALKBH4Ba	3221	511	6.08	57.38	nucleus
PagALKBH4Bb	3091	511	5.98	57.48	nucleus
PagALKBH5Ba	2190	269	5.45	30.53	nucleus
PagALKBH5Bb	3731	263	5.37	29.96	nucleus
PagALKBH6Ba	6847	525	5.72	56.96	nucleus
PagALKBH6Bb	6870	770	6	84.95	nucleus
PagALKBH7Ba	2029	259	4.62	29.28	nucleus
PagALKBH7Bb	1783	259	4.7	29.33	nucleus
PagFIP37a	7503	336	5.22	38.04	nucleus
PagFIP37b	6770	336	5.22	38.08	nucleus
PagHAKAI1	3511	498	6.06	53.75	nucleus
PagHAKAI2	3162	498	6.15	53.74	nucleus
PagMTA1	1294	454	6.24	49.99	nucleus
PagMTA2	1285	288	5.54	32.56	nucleus
PagMTB1	4948	1185	7.18	131.99	nucleus
PagMTB2	4755	1193	7.4	132.93	nucleus
PagMTB3	4711	1181	7.4	131.55	nucleus
PagMTB4	5307	1188	7.18	132.34	nucleus
PagMTC1	3822	406	6.58	46.93	nucleus
PagMTC2	3812	406	6.77	46.94	nucleus
PagVIRILIZER1	17,347	2179	5.26	238.44	nucleus
PagVIRILIZER2	16,715	2179	5.28	23.83	nucleus
PagECT1	3401	616	6.9	68.49	nucleus
PagECT2	6030	588	6.25	64.73	nucleus
PagECT3	4030	739	8.67	81.95	nucleus
PagECT4	4325	619	7.17	68.65	nucleus
PagECT5	3379	625	6.44	69.20	nucleus
PagECT6	4628	653	5.48	71.62	nucleus
PagECT7	5476	591	6.48	65.24	nucleus
PagECT8	5224	602	5.94	66.12	nucleus
PagECT9	5342	583	7.21	64.51	nucleus
PagECT10	6578	703	6.34	78.35	nucleus
PagECT11	7680	761	6.1	84.21	nucleus
PagECT12	4211	603	5.72	66.29	nucleus
PagECT13	3780	600	6.16	66.44	nucleus
PagECT14	4156	766	5.95	84.53	nucleus
PagECT15	4431	585	5.71	64.22	nucleus
PagECT16	4089	601	6.22	66.33	nucleus
PagECT17	5212	774	5.98	85.56	nucleus
PagECT18	5324	654	5.33	71.68	nucleus
PagECT19	5080	584	6.6	64.66	nucleus
PagECT20	4567	636	5.2	69.39	nucleus
PagECT21	9328	975	6.78	108.47	nucleus
PagECT22	4478	629	5.54	68.75	nucleus
PagECT23	4856	548	6.58	60.02	nucleus
PagECT24	6050	584	6.68	64.65	nucleus
PagECT25	4745	609	5.34	66.39	cytoplasm
PagECT26	4476	639	5.43	70.22	nucleus
PagECT27	4409	624	5.38	68.26	nucleus
PagECT28	4672	540	6.25	59.01	cytoplasm
PagCPSF30a	15,333	695	6.08	76.07	nucleus
PagCPSF30b	16,254	684	6.2	75.03	nucleus
PagCPSF30c	6063	398	6.17	44.82	nucleus
PagCPSF30d	15,158	684	6.2	74.99	nucleus
PagCPSF30e	3987	375	5.82	42.11	nucleus

**Table 2:** Amino acid residues participate in protein–ligand docking between LBD15 and predicted *cis*-elements in the promoters of m^6^A pathway genes.

Receptor Protein	Ligand	Predicted *Cis*-Element Sequence	Affinity (kcal/mol)	Amino Acid Residues in Docking	
				A Chain	B Chain
LBD15	PagFIP37b	CACCCGGAATTT	−5.3	Thr-44 Cys-56 Asn-103 Tyr-107 Arg-114	
LBD15	PagALKBH4Ba	AAACCGGAAAAG	−5	Arg-53	
LBD15	PagECT2	AAGCAGGAACTT	−4.8	Gln-129	Gln-128 Gln-132
LBD15	PagHAKAI2	CAGCAGGAGACA	−4.8	Arg-55 Ser-84	
LBD15	PagCPSF30a	CCCCCAGAAAAT	−4.7		Pro-45 Arg-55 Cys-56 Gln-58 Ser-63 Pro-64 Asp-100 Asn-103
LBD15	PagALKBH2Ba	CTGCCTGAGACA	−4.6	Leu-52 Arg-55 Glu-95	
LBD15	PagECT23	TCGCAGGCAATG	−4.5	Try-107 Asn-110	Gln-128
LBD15	PagECT13	GAGCTGGAAAAT	−4.4	Tyr-107	Asn-139 Ser-143
LBD15	PagECT15	GTTCCACCACCTG	−4.3	Glu-70 Asn-103 Aan-110	Ser-125
LBD15	PagECT28	TCGCAGGCAATG	−4.1	Thr-44 Glu-59 Cys-60 Lys-73 Ser-84	
LBD15	PagECT9	TCTCAGGAAACA	−4.1	Lys-73 Asn-110 Arg-114	
LBD15	PagECT16	TCTCCGCCGTCCC	−4.0	Phe-80 Ser-84	His-78 Asn-85
LBD15	PagECT7	AAACCGGAATAT	−3.7	His-69 Lys-73 Asn-103	Gln-128

**Table 3:** Protein–protein docking of PagMTAs, PagMTBs and PagMTCs. NHB: number of potential hydrogen bonds. NSB: number of potential salt bridges.

Structure 1	Structure 2	Interface	Inte	Inte	NHB	NSB
		Area, Å2	kcal/M	*p*-Value		
PagMTA1	PagMTC1	3151.8	−18.3	0.471	34	12
PagMTA1	PagMTC2	2655.1	−9.2	0.552	28	22
PagMTA1	PagMTB1	2326.9	−18.2	0.160	25	19
PagMTA1	PagMTB2	2321.8	−15.9	0.265	25	13
PagMTA1	PagMTB3	2326.7	−18.2	0.162	25	19
PagMTA1	PagMTB4	2321.8	−15.9	0.265	25	13
PagMTA2	PagMTC1	2613.1	−5.3	0.769	33	20
PagMTA2	PagMTC2	3360.1	−14.0	0.325	28	18
PagMTA2	PagMTB1	2326.9	−18.2	0.160	25	19
PagMTA2	PagMTB2	2321.8	−15.9	0.265	25	13
PagMTA2	PagMTB3	2326.7	−18.2	0.162	25	19
PagMTA2	PagMTB4	2321.8	−15.9	0.265	25	13
PagMTC1	PagMTB2	2335.9	−11.0	0.591	29	11
PagMTC2	PagMTB2	2391.4	−7.8	0.442	25	18
PagMTC1	PagMTB1	2281.4	−11.9	0.479	28	9
PagMTC2	PagMTB1	2445.5	−8.6	0.398	25	22
PagMTC1	PagMTB3	2391.4	−7.8	0.442	25	18
PagMTC2	PagMTB3	2281.1	−11.9	0.481	28	9
PagMTC1	PagMTB4	2281.8	−11.8	0.508	28	9
PagMTC2	PagMTB4	2391.4	−7.8	0.442	25	18

## Data Availability

The data in this study are available in the Supplementary Materials.

## Supplementary Materials

The following supporting information can be downloaded at: https://www.mdpi.com/article/10.3390/genes13061018/s1. Table S1: dn/ds analysis of writers, readers and erasers; Table S2: The cis-regulatory elements in the promoter of m6A pathway genes; Table S3: Accession numbers and amino acid sequences of m6A pathway genes from Arabidopsis thaliana and O.sativa; Table S4: The homology templates and docking score (RMSD) in protein homology modeling; Table S5: The primers used in qPCR; Figure S1: The results of molecular docking between cis-elements of m6A pathway genes and LBD15. The amino acid residues involved in docking are displayed in sticks and signed a label.
