# Supplementary material for: Genome-Wide Identification of m6A Writers, Erasers and Readers in Poplar 84K
Source: Genes (Basel). 2022 Jun 5;13(6):1018. doi: 10.3390/genes13061018 (PMC9223284; doi:10.3390/genes13061018)
Supplement: Supplementary file 1 [file genes-13-01018-s001.zip › genes-1718336-supplementary.pdf]

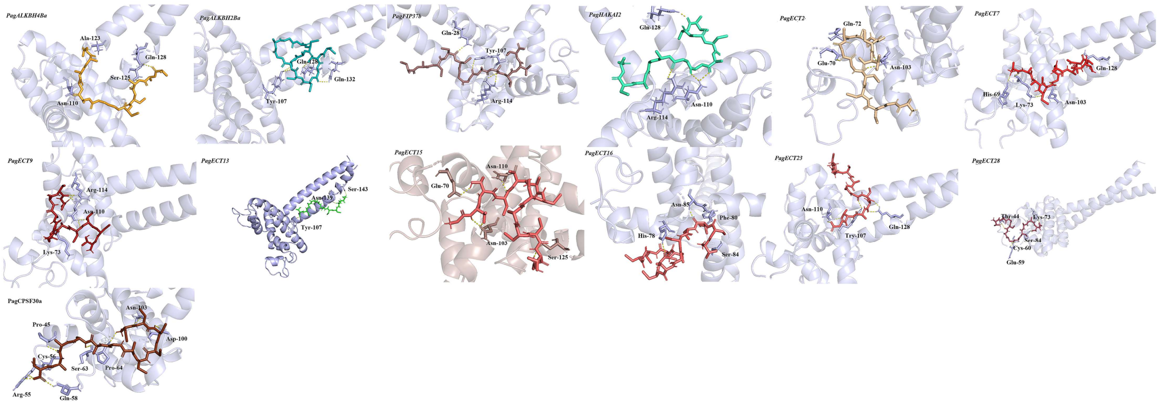

Figure S1. The results of molecular docking between *cis*-elements of m^{6}A pathway genes and *LBD15*.
